# Psychosocial Predictors of Non-Adherence and Treatment Failure in a Large Scale Multi-National Trial of Antiretroviral Therapy for HIV: Data from the ACTG A5175/PEARLS Trial

**DOI:** 10.1371/journal.pone.0104178

**Published:** 2014-08-25

**Authors:** Steven A. Safren, Katie B. Biello, Laura Smeaton, Matthew J. Mimiaga, Ann Walawander, Javier R. Lama, Aadia Rana, Mulinda Nyirenda, Virginia M. Kayoyo, Wadzanai Samaneka, Anjali Joglekar, David Celentano, Ana Martinez, Jocelyn E. Remmert, Aspara Nair, Umesh G. Lalloo, Nagalingeswaran Kumarasamy, James Hakim, Thomas B. Campbell, for the PEARLS (ACTG A5175) Study Team

**Affiliations:** 1 Massachusetts General Hospital, Boston, MA, United States of America; 2 Harvard Medical School, Boston, MA, United States of America; 3 The Fenway Institute, Fenway Health, Boston, MA, United States of America; 4 Harvard School of Public Health, Boston, MA, United States of America; 5 Frontier Science and Technology Research Foundation, Amherst, NY, United States of America; 6 Asociacion Civil Impacta Salud y Educacion, Lima, Peru; 7 Alpert Medical School/The Miriam Hospital, Providence, RI, United States of America; 8 College of Medicine – Johns Hopkins Research Project, Blantyre, Malawi; 9 University of North Carolina Project, Lilongwe, Malawi; 10 University of Zimbabwe-University of California San Francisco Collaborative Research Program, Harare, Zimbabwe; 11 National AIDS Research Institute, Pune, India; 12 Johns Hopkins Bloomberg School of Public Health, Baltimore, MD, United States of America; 13 NIH/NIAD/DAIDS Pharmaceutical Affairs, Bethesda, MD, United States of America; 14 Nelson R Mandela School of Medicine, University of KwaZulu-Natal, Durban, S. Africa; 15 YRGCARE Medical Centre, Chennai, India; 16 University of Zimbabwe, Harare, Zimbabwe, Africa; 17 University of Colorado Denver, Aurora, CO, United States of America; UCL Institute of Child Health, University College London, United Kingdom

## Abstract

**Background:**

PEARLS, a large scale trial of antiretroviral therapy (ART) for HIV (n = 1,571, 9 countries, 4 continents), found that a once-daily protease inhibitor (PI) based regimen (ATV+DDI+FTC), but not a once-daily non-nucleoside reverse transcriptase inhibitor/nucleoside reverse transcriptase inhibitor (NNRTI/NRTI) regimen (EFV+FTC/TDF), had inferior efficacy compared to a standard of care twice-daily NNRTI/NRTI regimen (EFV+3TC/ZDV). The present study examined non-adherence in PEARLS.

**Methods:**

Outcomes: non-adherence assessed by pill count and by self-report, and time to treatment failure. Longitudinal predictors: regimen, quality of life (general health perceptions  =  QOL-health, mental health  =  QOL-mental health), social support, substance use, binge drinking, and sexual behaviors. “Life-Steps” adherence counseling was provided.

**Results:**

In both pill-count and self-report multivariable models, both once-a-day regimens had lower levels of non-adherence than the twice-a-day standard of care regimen; although these associations attenuated with time in the self-report model. In both multivariable models, hard-drug use was associated with non-adherence, living in Africa and better QOL-health were associated with less non-adherence. According to pill-count, unprotected sex was associated with non-adherence. According to self-report, soft-drug use was associated with non-adherence and living in Asia was associated with less non-adherence. Both pill-count (HR = 1.55, 95% CI: 1.15, 2.09, p<.01) and self-report (HR = 1.13, 95% CI: 1.08, 1.13, p<.01) non-adherence were significant predictors of treatment failure over 72 weeks. In multivariable models (including pill-count or self-report nonadherence), worse QOL-health, age group (younger), and region were also significant predictors of treatment failure.

**Conclusion:**

In the context of a large, multi-national, multi-continent, clinical trial there were variations in adherence over time, with more simplified regimens generally being associated with better adherence. Additionally, variables such as QOL-health, regimen, drug-use, and region play a role. Self-report and pill-count adherence, as well as additional psychosocial variables, such QOL-health, age, and region, were, in turn, associated with treatment failure.

## Background

Antiretroviral therapy is increasingly available in diverse parts of the world [1], with many high-prevalence settings providing antiretroviral therapy for those who meet country-specific criteria. High levels of sustained life-long adherence are required for the successful treatment of HIV, which in turn, can prevent HIV progression. Additionally, wide-scale successful treatment for HIV may decrease HIV incidence in endemic settings, as it has been shown that early HIV treatment can prevent HIV transmission in serodiscordant couples [2].

Although adherence in resource poor settings may actually be equal to or better than in North American settings [3]–[5] in 2006, 12 studies from Africa were available for a meta-analysis, which suggested an estimate of 77% of populations achieving adequate levels of adherence [3]. Accordingly, at that time, there was a significant minority of individuals not achieving optimal adherence. Several qualitative and quantitative studies have examined factors related to non-adherence in diverse settings, finding associations with variables such as social supports, side effects, lack of/inadequate counseling, stigma by healthcare workers in Tanzania [6]; socio-economic factors, patient/family variables, healthcare systems in Africa [7]; depression and negative attitudes about antiretroviral therapy (ART) in Haiti [8]; younger age, greater pill burden, higher number of doses per day, race, protease inhibitor (PI)-based regimens in a large scale international trial [9]; stigma to self or family, mental health, economic problems, drug use in China [10]; and non-disclosure of HIV status, female sex, being illiterate, side effects, being on ART for fewer than 2 years, alcohol use, travel time, and lack of knowledge and negative perceptions about ART in Nepal [11].

The Prospective Evaluation of Antiretrovirals in Resource Limited Settings (PEARLS) study (ACTG5175) [12] was a large-scale, randomized clinical non-inferiority trial studying 1,571 HIV-infected individuals from 9 countries and 4 continents (34.7% Africa, 29.5% Latin America/Carribean, 22.6% Asia, and 13.3% United States) with the goal of understanding if simplified regimens (once daily) could perform as well as a standard of care twice-daily regimen. The primary findings were that a non-nucleoside reverse transcriptase inhibitor/nucleoside reverse transcriptase inhibitor (NNRTI/NRTIs) regimen, consisting of efavirenz plus co-formulated emtricitabine-tenofovir-DF (EFV+FTC/TDF), for which all components were dosed once daily at the same time, had similar efficacy to a standard of care NNRTI/NRTIs regimen consisting of efavirenz plus co-formulated lamivudine-zidovudine, (EFV+3TC/ZDV), where the 3TC/ADV are NRTI components taken twice daily, but that a protease-inhibitor (PI) regimen of atazanavir plus didanosine-EC and emtricitabine (ATV+DDI+FTC), in which all components were dosed once daily, but the DDI and ATV components were at different times, was inferior to the standard of care regimen. This study contained a brief psychosocial assessment battery, allowing for a longitudinal examination of whether the regimens in which all components were dosed once daily (EFV+FTC/TDF and AVT+DDI+FTC) would have lower non-adherence than the standard of care regimen that requires twice a day dosing of the NRTI components, and whether additional predictors of adherence would be relevant in a diverse international cohort of individuals starting ART.

## Methods

Details of the overall study design can be found both on clinicaltrials.gov NCT00084136 and in the primary outcome paper [12]. Study sites were as follows: Instituto de Pesquisa Clinica Evandro Chagas, Rio de Janeiro, Brazil; Hospital Nossa Senhora da Conceicao-GHC, Porto Alegre, Brazil; Les Centres GHESKIO, Port-au-Prince, Haiti; YRG Centre for AIDS Research & Education, Chennai, India; National AIDS Research Institute, Pune, India; College of Medicine Clinical Research Site, Blantyre, Malawi; Kamuzu Central Hospital, Lilongwe, Malawi; Asociacion Civil Impacta Salud y Educacion - Miraflores and San Miguel Clinical Research Site, Lima, Peru; Durban Adult HIV Clinical Research Site, Durban, South Africa; University of Witwaterself-reportand Clinical HIV Research Unit, Johannesburg, South Africa; Research Institute for Health Sciences, Chiang Mai, Thailand; and Parirenyatwa Hospital Clinical Research Center, Harare, Zimbabwe. All ACTG sites in the United States were also eligible to enroll participants. Each individual site's own IRB or equivalent organization approved this study. Enrollment in the US was limited to no more than 18% of total; the remaining enrollment was distributed equally across the international sites with an option for international sites to request additional enrollment once their initial quota of 100 participants was filled. Participants gave written informed consent to participate in this study.

To be included in the study, participants needed to be at least 18 years old, have documented HIV-1 infection, have CD4+ lymphocytes below 300 cells/mm^3^ and have not had ART previously (i.e. no more than 7 days of cumulative antiretroviral therapy prior to study entry, with exception of ZDV or single dose nevirapine for pMTCT use). Exclusion criteria generally were if any of the medication regimens was medically contra-indicated. Women of reproductive potential who were non-pregnant and, if participating in sexual activity that could lead to pregnancy, agreed to use contraception (two forms if taking EFV). Participants were randomized 1∶1∶1 to an open-label regimen of efavirenz 600 mg daily plus co-formulated lamivudine-zidovudine 150mg/300 mg twice daily (EFV+3TC-ZDV); or atazanavir 400 mg once daily with food, plus didanosine-EC 400 mg once daily taken on an empty stomach 1 hour before or 2 hours after the atazanavir dose, plus emtricitabine 200 mg once daily (ATV+DDI+FTC); or efavirenz 600 mg once daily plus co-formulated emtricitabine-tenofovir-DF 200 mg/300 mg once daily (EFV+FTC-TDF).

Participant flow has been described previously [12]. In brief, 1571 participants were randomized, and 99% of expected study visits were completed. Although the parent trial continued follow-up for participants who failed the first antiretroviral regimen, the present analysis only included data up to regimen failure, with a maximum of 72 weeks, which was the median time point for when the Data Safety Monitoring Board recommended stopping the ATV+DDI+FTC arm due to inferiority.

### Measures

Participants completed an interviewer-administered adherence questionnaire (ACTG QOL0061) and pill counts at every study visit which yielded a non-adherence self-report score and a non-adherent pill-count categorization. These were at weeks 2, 4, 8, 12, 16, 20, 24, 32, 40, 48 since start of regimen and every 8 weeks through end of follow up. A longer psychosocial interview (ACTG QOL0060) was administered at entry, and weeks 16, 32, and 48 since start of regimen and every 48 weeks through end of follow up. At all sites, self-report measures were translated and back-translated to maximize accuracy, and administered in a face to face interview by study nurses in the local language. These measures are described below.

#### Adherence questionnaire

The adherence questionnaire started with a grid whereby each study drug was listed with the number of doses prescribed per day filled in by study staff. Participant would answer the number or prescribed doses missed for “yesterday”, “2 days ago”, “3 days ago” and “the past two weeks”. They were then asked a series of questions including when they last missed medications (within past week, 1–2 weeks ago, 2–4 weeks ago, 1–3 months ago, more than three months ago, or never missed/n.a.), how many days they had missed taking all of their doses during the past four days (none, one day, two days, three days, four days), and whether they missed any medications over the past weekend (yes/no for Saturday OR Sunday). These 7 questions were scored such that they constituted a non-adherence score, as described in the data analysis section below. Similar self-report questions with different recall periods have been used in adherence assessment [3], [5], [9] and intervention [13] studies in diverse settings such as these.

Additionally, participants were asked about potential reasons for non-adherence. This involved a checklist for “never”, “rarely” “sometimes” and “often” and had 24 potential reasons for non-adherence such as “forgot”, “side effects” “transportation problems getting to the clinic”, “lost pills” which were generated from the study sites and using items from prior ACTG trials [14]. The most frequently reported reasons are described.

#### Pill Count

At each study visit, participants were instructed to bring any remaining pills to the clinic for a pill count. Study nurses counted pills expected which yielded a binomial pill count non-adherence score (missed any pills versus did not miss any pills). If participants forgot their medicines, these data were coded as missing.

#### Psychosocial Interview

The psychosocial interview included a modified version [15] of the ACTG SF-21 [16]. To simplify the analyses, and based on prior adherence research, only the general health perceptions and mental health subscales were included. To aid interpretation, this was scaled on to 1–10. We also included one ACTG question about general satisfaction with social support [14], [15], [17]; which asked about overall satisfaction with social support from friends and families, ranging from 0 (very dissatisfied) to 3 (Very satisfied). For substance use, there was a frequency question about binge drinking, asking how often participants drank 5 or more drinks of alcohol in the past month, ranging from never (0), to daily (6), a series of yes/no questions for various hard drugs (e.g. cocaine, heroin) and marijuana (considered soft drug use), followed by the frequency question for the substance used most. Lastly, there were questions about sexual behaviors in the past month, which yielded a variable indicating whether or not participants reported any HIV sexual transmission risk behavior in the past week.

#### Treatment Failure

Treatment failure was defined according to ACTG protocol [12]. Briefly, it was defined as two successive measurements of plasma HIV-1 RNA> = 1000 copies/mL with the first measurement at the week 16 visit or later, disease progression at the week 12 visit or later, or death, regardless of study treatment history or status (intention-to-treat).

### Data Analysis

For the self-report non-adherence score, we followed the methodology of Reynolds et al. (2007) [18] and performed principal component (PC) analysis using the adherence questions described above to construct a non-adherence factor, approximating a latent variable. This PC analysis was conducted for each visit separately. We retained one PC because for each visit, with only one exception, only the first PC had an eigenvalue greater than 1 [19], and the results of a screen test also suggested that only the first component was meaningful [20]. Additionally, the majority (>50%) of the variance was explained by the first PC for each visit.

Overall frequencies for categorical variables and means for continuous measures were calculated. Additionally, reasons for non-adherence (among those who reported being non-adherent) were described using frequencies at the first visit after initiation (week 2). Because three participants did not complete a baseline questionnaire, they were not included in the baseline summary measures, resulting in an analytic sample size of 1,568.

For risk factors of non-adherence over time, unadjusted mixed-effects regressions were calculated using PROC GLIMMIX (for missed pills) and PROC MIXED (for non-adherence PC score) in SAS v. 9.3. All models included a random intercept with an unstructured covariance, and all models included "month of follow up" as a main effect. The effect of treatment condition on change in non-adherence was examined by including the treatment by time interaction term in both models. Factors associated with non-adherence over time at p = 0.10 were included in multivariable, adjusted regression models. Mixed-effects regressions allow for unbalanced and missing data; however, because there was no data on psychosocial variables at certain measurement occasions by design (i.e. some variables assessed every weeks and others every 4 months), we performed “last observation carried forward” on these measures so that the these measurement occasions could be included – as a result data from all 1,571 participants contributed to the analysis.

Finally, in order to examine risk factors for week to treatment failure, unadjusted Cox proportional hazard regressions were used. All factors associated with week to treatment failure at p = 0.10 were included in adjusted, multivariable Cox Proportional Hazard (PH) regressions. Additionally, treatment condition was included but not reported in this analysis, as treatment effects have been reported previously [12]. Cox regressions require complete-case data analysis. Because the psychosocial risk factors were not measured at every measurement occasion by design, we again performed “last observation carried forward” on these measures. The analytic sample for factors associated with treatment failure was 1,571. However, a complete-case analysis was performed for unadjusted and adjusted regressions.

## Results

Demographic variables and baseline mean and standard deviation scores are presented in Table 1. At initiation (week 2), 16.0% (N = 187) of participants missed at least one pill via pill count (13.8%, 14.8%, and 19.5% for the once-daily PI-based regimen, once-daily NNRTI-based regimen, and twice-daily standard of care regimen, respectively). According to the relevant self-report item 11.5% (N = 174) of participants reported having missed at least one pill in the past 2 weeks (8.4%, 11.0%, and 15.1% for the once-daily PI-based regimen, once-daily NNRTI-based regimen, and twice-daily standard of care regimen, respectively).

**Table 1:** Baseline Participant Characteristics.

	Overall (N = 1568)
**Categorical Variables**	
Age	
<25	135 (8.6)
25–<30	298 (19.0)
30–<35	371 (23.7)
35–<40	317 (20.2)
40–<45	227 (14.5)
> = 45	220 (14.0)
Sex	
Female	739 (47.1)
Male	829 (52.9)
Region	
Latin America/Caribbean	464 (29.6)
Asia	355 (22.6)
Africa	540 (34.4)
United States	209 (13.3)
Binge Alcohol Use	
At least weekly	108 (6.9)
Less than weekly	1455 (93.1)
Soft Drug Use, past month	
Yes	48 (3.1)
No	1501 (96.9)
Hard Drug Use, past month	
Yes	20 (1.3)
No	1530 (98.7)
Any Unprotected Sex, past month	
Yes	68 (4.6)
No	1402 (95.4)
Satisfaction with Social Support	
Very satisfied	1019 (65.4)
Somewhat satisfied	375 (24.1)
Very/somewhat dissatisfied	165 (10.6)
**Continuous Measures**	**Mean (SD)**
QOL Subscales (0–10), mean (SD)	
QOL_health	5.99 (2.45)
QOL_mental	5.71 (1.31)
**Categorical Measures**	**N (%)**
Treatment	
Once daily PI+ NRTIs: ATV+DDI+FTC	524 (33.4)
Once daily NNRTI + NRTsI: EFV+FTC- TDF	525 (33.5)
Standard of care:	519 (33.1)
EFV+3TC-ZDV	

Table Legend:

QOL_health: general health perceptions.

QOL_mental: mental health.

Treatment: Once daily protease inhibitor + nucleoside reverse transcriptase inhibitors: atazanavir + didanosine-EC and emtricitabine.

Treatment: Once daily non-nucleoside reverse transcriptase inhibitor + nucleoside reverse transcriptase inhibitors: efavirenz + co-formulated emtricitabine-tenofovir-DF.

Standard of care: efavirenz plus co-formulated lamivudine-zidovudine.

### Reasons for non-adherence, initiation (week 2)

Among those who reported missing any doses of any study-prescribed medication in the past 2 weeks at initiation, the most common reasons endorsed were forgetting to take pills (37.4%) and to avoid side effects (33.3%). Additionally, more than 10% endorsed the following reasons for non-adherence: bad events from pills (12.6%), did not understand the regimen (11.5%), travel away from home (10.9%), and other illness got in the way (10.3%). No one endorsed the following reasons for non-adherence: shared ART with family or friends, religious beliefs, ran out of pills, tired of taking so many pills, pills got damaged, too ill to pick up pills, or pills were stolen.

### Longitudinal models of non-adherence: Weeks 2 through 72

#### Bivariate association with time

Pill count non-adherence increased over time (p<0.01); however, there was no significant change in self-report non-adherence over time (p = 0.23) (Table 2).

**Table 2:** Longitudinal Bivariate Predictors of Non-Adherence (Missed any pills since last visit)^1^^,^ 2.

	Pill Count			Self Report		
	OR	95% CI	p-value	Parameter Estimate	95% CI	p-value
Regimen			<0.01			<0.01
1-once daily PI+ NRTIs: ATV+DDI+FTC	0.72	(0.60, 0.86)	<0.01	−0.16	(−0.27, −0.05)	<0.01
2-once daily NNRTI + NRTIs EFV+FTC-TDF	0.53	(0.44, 0.63)	<0.01	−0.16	(−0.27, −0.06)	<0.01
3-standard of care: EFV+3TC-ZDV	1.00			0.00		-
Time (Month)	1.05	(1.04, 1.06)	<0.01	0.003	(−0.002, 0.01)	0.23
QOL Subscales						
QOL_health	0.95	(0.93, 0.98)	<0.01	−0.04	(−0.06, −0.03)	<0.01
QOL_mental	0.97	(0.93, 1.01)	0.20	−0.05	(−0.08, −0.02)	<0.01
Age			0.23			0.49
<25	1.37	(1.00, 1.88)	-	0.13	(−0.06, 0.32)	-
25−<30	1.16	(0.90, 1.51)	-	0.10	(−0.05, 0.26)	-
30−<35	1.08	(0.84, 1.38)	-	0.05	(−0.10, 0.20)	-
35−<40	1.24	(0.97, 1.61)	-	0.09	(−0.06, 0.25)	-
40−<45	1.01	(0.76, 1.33)	-	−0.01	(−0.18, 0.15)	-
> = 45	1.00			0.00		
Female Sex	0.74	(0.64, 0.86)	<0.01	−0.23	(−0.32, −0.14)	<0.01
Region			<0.01			<0.01
Latin America/Caribbean	0.92	(0.75, 1.14)	0.46	−0.21	(−0.35, −0.06)	<0.01
Asia	0.78	(0.61, 0.98)	0.03	−0.38	(−0.53, −0.23)	<0.01
Africa	0.52	(0.42, 0.64)	<0.01	−0.55	(−0.69, −0.40)	<0.01
United States	1.00			0.00		
Binge Alcohol Use (at least weekly)	1.35	(1.09, 1.68)	<0.01	0.13	(−0.02, 0.28)	0.08
Soft Drug Use, past month	1.48	(1.05, 2.08)	0.02	0.53	(0.30, 0.76)	<0.01
Hard Drug Use, past month	2.85	(1.74, 4.66)	<0.01	0.92	(0.54, 1.31)	<0.01
Any Unprotected Sex, past month	1.48	(1.13, 1.95)	<0.01	0.19	(−0.002, 0.38)	0.05
Satisfaction with Social Support			0.23			<0.01
Very satisfied	0.85	(0.71, 1.02)	-	−0.21	(−0.34, −0.09)	<0.01
Somewhat satisfied	0.87	(0.72, 1.06)	-	−0.16	(−0.29, −0.02)	0.02
Very/somewhat dissatisfied	1.00			0.00		

1 All models include random intercept (covariance = unstructured).

2 All models include "month of follow up" as a main effect.

Table Legend:

Regimen 1: Once daily protease inhibitor + nucleoside reverse transcriptase inhibitors: atazanavir + didanosine-EC and emtricitabine.

Regimen 2: Once daily non-nucleoside reverse transcriptase inhibitor + nucleoside reverse transcriptase inhibitors: efavirenz + co-formulated emtricitabine-tenofovir-DF.

Regimen 3: Standard of care: efavirenz plus co-formulated lamivudine-zidovudine.

QOL_health: general health perceptions.

QOL_mental: mental health.

#### Bivariate predictors of non-adherence Weeks 2 through 72

Table 2 presents main effects bivariate predictors of self-report and pill count non-adherence. For both pill-count and self-report, both once daily regimens were associated with lower levels of non-adherence than the twice-daily standard of care regimen. For self-report only, however, the main effect for time was qualified by a significant interaction (p = 0.01). Specifically, the associations between the once-daily NNRTI/NRTIs regimen (EFV+FTC/TDF) and the PI-based regimen (ATV+DDI+FTC) and self-report non-adherence was attenuated over follow up when compared to the standard of care regimen. For example, at month 1, the once-daily PI-based regimen (Est. = −0.27, p<0.01) was significantly associated with lower non-adherence than the standard of care regimen. However, by month 10, the once-daily PI-based regimen (Est. = −0.09, p = 0.11) was no longer associated with non-adherence when compared to the standard of care regimen.

For both pill-count and self-report non-adherence, better QOL general health perceptions and female sex were also associated with lower likelihood of non-adherence. In both pill-count and self-report, region was a significant predictor of non-adherence, with Africa and Asia having lower levels of non-adherence than the U.S. referent group, and for self-report, Latin-America having lower levels of non-adherence than the U.S. Soft and hard drug use and any unprotected sex were associated with higher non-adherence for both outcomes. For just the pill-count non-adherence indicator, binge alcohol use was associated with higher levels of non-adherence. Better QOL mental health and more satisfaction with social support were associated with lower self-report non-adherence.

#### Multivariable models of non-adherence from weeks 2 through 72 (Table 3)

Per the plan for selecting variables from the bivariate models (via meeting the criterion of having a p value of <0.10), both multivariable models (pill count and self-report) included month of follow up, QOL-health subscale, treatment regimen, sex, region, binge alcohol use, soft drug use, hard drug use, and any unprotected sex as potential risk factors. The self-report model also included QOL-mental health and satisfaction with social support.

**Table 3:** Longitudinal Multivariable Predictors of Non-Adherence^1^.

	Pill Count			Self Report		
	aOR	95% CI	p-value	Parameter Estimate	95% CI	p-value
Month	1.06	(1.05, 1.06)	<0.01	0.008	(0.002, 0.01)	<0.01
QOL Subscales						
QOL_health	0.95	(0.93, 0.97)	<0.01	−0.04	(−0.05, −0.02)	<0.01
QOL_mental	—	—	—	−0.03	(−0.06, −0.00)	0.04
Treatment			<0.01			<0.01
1-once daily PI + NRTIs: ATV+DDI+FTC	0.72	(0.61, 0.86)	<0.01	−0.14	(−0.25, −0.04)	<0.01
2-once daily NNRTI + NRTIs: EFV+FTC-TDF	0.52	(0.43, 0.62)	<0.01	−0.16	(−0.27, −0.05)	<0.01
3-standard of care: EFV+3TC-ZDV	1.00			0.00		
Female Sex	0.88	(0.75, 1.02)	0.09	−0.12	(−0.21, −0.03)	0.01
Region			<0.01			<0.01
Latin America/Caribbean	0.97	(0.76, 1.23)	0.79	−0.08	(−0.23, 0.07)	0.31
Asia	0.87	(0.67, 1.12)	0.28	−0.21	(−0.37, −0.05)	0.01
Africa	0.55	(0.43, 0.71)	<0.01	−0.39	(−0.55, −0.24)	<0.01
United States	1.00			0.00		
Binge Alcohol Use (at least weekly)	1.21	(0.97, 1.50)	0.09	0.03	(−0.12, 0.18)	0.74
Soft Drug Use, past month	1.11	(0.78, 1.58)	0.57	0.28	(0.05, 0.52)	0.02
Hard Drug Use, past month	2.16	(1.28, 3.62)	<0.01	0.70	(0.29, 1.11)	<0.01
Any Unprotected Sex, past month	1.47	(1.12, 1.93)	<0.01	0.15	(−0.03, 0.34)	0.12
Satisfaction with Social Support			—			0.07
Very satisfied	—	—	—	−0.15	(−0.27, −0.02)	—
Somewhat satisfied	—	—	—	−0.15	(−0.29, −0.01)	—
Very/somewhat dissatisfied	—			0.00		

1 Model includes random intercept (covariance = unstructured).

Table Legend:

QOL_health: general health perceptions.

QOL_mental: mental health.

Treatment 1: Once daily protease inhibitor + nucleoside reverse transcriptase inhibitors: atazanavir + didanosine-EC and emtricitabine.

Treatment 2: Once daily non-nucleoside reverse transcriptase inhibitor + nucleoside reverse transcriptase inhibitors: efavirenz + co-formulated emtricitabine-tenofovir-DF.

Treatment 3: Standard of care: efavirenz plus co-formulated lamivudine-zidovudine.

In both self-report non-adherence and pill-count non-adherence models, both once-daily treatment regimens were associated with lower levels of non-adherence. Additionally, QOL general health perceptions and living in Africa (compared to the U.S.) were associated with lower non-adherence with both indicators of non-adherence. Hard-drug use was associated with higher non-adherence for both indicators of non-adherence.

For just the pill-count model, any unprotected sex in the past month was associated with higher non-adherence.

For just the self-report model, QOL-mental health and female sex were associated with lower non-adherence, and soft-drug use was associated with higher non-adherence.

Additionally, for self-report only, there was an interaction for the effect of treatment regimen with time on non-adherence (p = 0.02) such that the associations between both once-daily regimens and self-report non-adherence were attenuated over follow up when compared to the standard of care regimen. Specifically, at month 1, when compared to the standard of care regimen, the once-daily PI-based regimen (parameter est. = −0.24, p<0.01) and the one daily NNRTI/NRTIs regimen (parameter est. = −0.16, p = 0.01) were both significantly associated with lower non-adherence than standard of care. However, by month 10, the once-daily PI-based regimen (parameter est. = −0.09, p = 0.13) was no longer associated with non-adherence, but the once daily NNRTI/NRTIs regimen remained associated with lower non-adherence (parameter est. = −0.16, p<0.01). By the end of follow up, neither once-daily regimen was significantly associated with non-adherence (p>0.05) when compared to the standard-of-care regimen, as the level of non-adherence for the PI-based regimen increased at a greater slope than the standard of care regimen throughout the follow-up. The once daily NNRTI/NRTIs regimen also increased in non-adherence, but at a similar rate as standard of care, thus continuing to have lower non-adherence than the standard of care.

### Predictors of time to treatment failure

Table 4 includes risk factors for time to treatment failure in four different approaches. The first set of models (unadjusted analyses) consists of unadjusted analyses. Next, we built a model with the psychosocial and demographic variables together, but not the non-adherence measures, then models including pill count non-adherence or self-report non-adherence, respectively.

**Table 4:** Risk Factors of Time to Treatment Failure.

	Unadjusted			Adjusted (no adherence)^1^			Adjusted (with pill count)^1^			Adjusted (with self-report score)^1^		
	HR	95% CI	p-value	HR	95% CI	p-value	HR	95% CI	p-value	HR	95% CI	p-value
QOL Subscales												
QOL_health	0.85	(0.81, 0.90)	<0.01	0.88	(0.82, 0.93)	<0.01	0.88	(0.83, 0.93)	<0.01	0.88	(0.83, 0.94)	<0.01
QOL_mental	0.86	(0.77, 0.95)	<0.01	1.01	(0.90, 1.14)	0.95	1.01	(0.90, 1.14)	0.89	1.00	(0.89, 1.13)	0.99
Age			0.07			0.02			0.03			0.04
<25	1.69	(0.94, 3.04)	0.08	1.97	(1.09, 3.54)	0.02	1.91	(1.06, 3.45)	0.03	1.83	(1.01, 3.31)	0.05
25−<30	1.95	(1.20, 3.16)	<0.01	2.21	(1.36, 3.60)	<0.01	2.11	(1.29, 3.44)	<0.01	2.04	(1.24, 3.35)	<0.01
30−<35	1.43	(0.88, 2.33)	0.15	1.61	(0.98, 2.63)	0.06	1.56	(0.95, 2.55)	0.08	1.57	(0.95, 2.59)	0.08
35−<40	1.38	(0.83, 2.28)	0.21	1.57	(0.94, 2.60)	0.08	1.51	(0.91, 2.51)	0.11	1.45	(0.86, 2.44)	0.16
40−<45	1.17	(0.67, 2.04)	0.58	1.21	(0.69, 2.11)	0.51	1.17	(0.67, 2.04)	0.59	1.13	(0.64, 2.00)	0.67
> = 45	1.00			1.00			1.00			1.00		
Female Sex	0.94	(0.72, 1.21)	0.62	—	—	—	—	—	—	—	—	—
Region			<0.01			<0.01			<0.01			<0.01
Latin America/Caribbean	0.33	(0.22, 0.49)	<0.01	0.35	(0.23, 0.53)	<0.01	0.37	(0.24, 0.55)	<0.01	0.41	(0.27, 0.62)	<0.01
Asia	0.37	(0.39, 1.02)	<0.01	0.37	(0.24, 0.56)	<0.01	0.38	(0.25, 0.59)	<0.01	0.44	(0.28, 0.68)	<0.01
Africa	0.64	(0.46, 0.89)	<0.01	0.62	(0.44, 0.88)	<0.01	0.68	(0.48, 0.96)	0.03	0.78	(0.55, 1.11)	0.17
United States	1.00			1.00			1.00			1.00		
Binge Alcohol Use (at least weekly)	0.84	(0.44, 1.58)	0.58	—	—	—	—	—	—	—	—	—
Soft Drug Use, past month	0.74	(0.30, 1.79)	0.50	—	—	—	—	—	—	—	—	—
Hard Drug Use, past month	0.62	(0.09, 4.40)	0.63	—	—	—	—	—	—	—	—	—
Any Unprotected Sex, past month	0.67	(0.25, 1.80)	0.42	—	—	—	—	—	—	—	—	—
Satisfaction with Social Support			<0.01			0.29			0.34			0.33
Very satisfied	0.55	(0.36, 0.85)	<0.01	0.80	(0.51, 1.24)	—	0.82	(0.52, 1.28)	—	0.83	(0.52, 1.30)	—
Somewhat satisfied	0.77	(0.48, 1.25)	0.29	1.00	(0.62, 1.63)	—	1.02	(0.62, 1.65)	—	1.04	(0.64, 1.71)	—
Very/somewhat dissatisfied	1.00			1.00			1.00					
Non-adherence Measures												
Missed any pills, since last visit	1.55	(1.15, 2.09)	<0.01	—	—	—	1.47	(1.08, 2.00)	0.01			
Self-reported non-adherence score	1.10	(1.08, 1.13)	<0.01	—	—	—	—	—	—	1.10	(1.07, 1.12)	<0.01

1 All adjusted models include treatment condition.

Table Legend:

QOL_health: general health perceptions.

QOL_mental: mental health.

#### Unadjusted analyses

In the first, unadjusted set of analyses, lower scores on both QOL subscales, specific region (being from the U.S. compared to other regions), lower levels of satisfaction with social support, and both non-adherence measures were associated with higher hazard of treatment failure within the first 72 weeks of follow-up. The association between age and non-adherence was marginally significant (p = .07).

#### Multivariable analyses with psychosocial variables

In the second approach, which included the psychosocial and demographic variables, but not non-adherence measures, lower QOL health scores, younger age (<25 and 25–30 versus > = 45), and specific regions (being from the U.S. compared to other regions) remained having higher hazard of treatment failure within the first 72 weeks of follow-up.

#### Multivariable model adjusting for pill-count nonadherence

**Figure 1:**
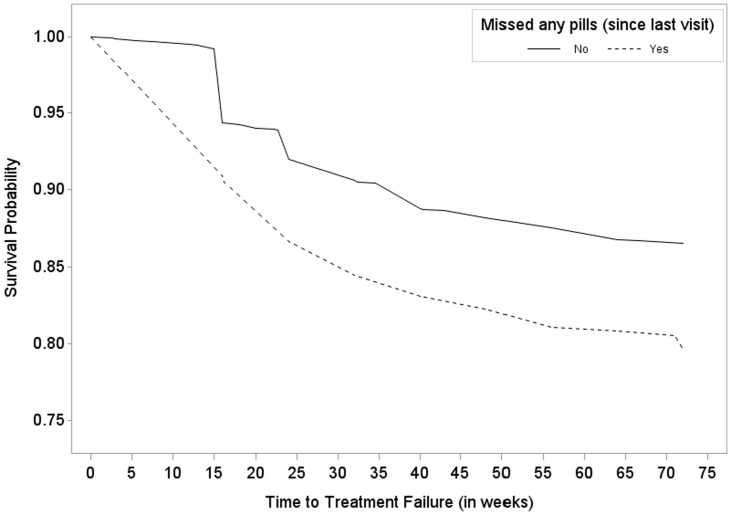
ACTG A5175/Pearls trial – Survival probability estimate from randomization to treatment failure by pill count non-adherence (solid line  =  did not miss any pills; dashed line  =  missed any pills).

Lower QOL health scores, younger age (<25 and 25–30 versus > = 45), region (being from the U.S. compared to other regions), and having missed any pills via pill count were associated with higher hazard of treatment failure within first 72 weeks of follow-up. Because pill-count non-adherence is a categorical variable, survival curves can be graphically portrayed for time to treatment failure, stratified by adherent versus non-adherent. This is depicted in Figure 1.

#### Multivariable model adjusting for self-report non-adherence

Lower QOL health scores, younger age (<25 and 25–30 versus > = 45), region (being from the U.S. compared to Latin America and Asia), and higher self-report non-adherence scores were associated with higher hazard of treatment failure within the first 72 weeks of follow-up.

## Discussion

With ART as a transformative treatment for HIV for over 20 years, an emphasis of refining treatment regimens is on simplifying patient burden with respect to dosing, whilst making ART available globally. Although one might expect that once a day regimens would be easier for adherence versus more frequent dosing, this has received minimal empirical study in low- and middle-income countries. With respect to efficacy, the primary outcome of the PEARLS study showed noninferiority of a once-daily NNRTI/NRTIs regimen compared to standard of care. The present study follows up on that finding, revealing that overall both once-daily regimens generally had lower pill-count and self-report non-adherence than the standard of care twice a day dosing. For example, according to pill-count, these two regimens had 28 and 47 percent lower odds of non-adherence when averaged over follow up. Note that the PI-based regimen was shown in the primary outcome trial to be inferior to standard of care, and hence the lower non-adherence in the PI-based regimen compared to standard of care suggests that the primary inferiority finding was not due to poor adherence but instead worse virologic potency. Pill count assessed non-adherence increased over time, and this change over time did not vary by regimen: for pill-count both once-daily regimens had lower non-adherence than standard of care. Self-report non-adherence also increased over time as a main effect, but its association with regimen did change with time. For both once-daily regimens compared to the standard of care regimen, the associations with lower self-report non-adherence waned with time. It is unclear as to why this pattern of results occurred. Examining the slopes of self-report non-adherence, all groups increased non-adherence over time, with the PI-based regimen having the fastest rate of worsening adherence. Additionally, the standard of care regimen also had increased risk for safety events in the primary trial, and this also could have contributed to higher self-reported non-adherence as time went on. It is unclear, however, why this happened with the self-report and not the pill-count outcome, so this should be interpreted as preliminary.

In the present study, after accounting for regimen, both pill-count and self-report non-adherence was associated with earlier treatment failure. Taken together, these data would suggest that once-a-day dosing, in the context of similar efficacy, may result in lower non-adherence and better outcomes in individuals receiving ART for HIV.

Additional findings from this analysis revealed that certain psychosocial variables were associated with non-adherence in multivariable models. These findings are generally consistent with existing studies of adherence in global settings referenced above, highlighting the importance of the psychosocial context of health behavior in multiple settings. For example, lower quality of life mental health scores were associated with higher non-adherence in the self-report bivariate and multivariable models. This is a potentially modifiable variable through counseling or referrals in that providers can potentially help patients address such problems. Lower general health perceptions were associated with higher non-adherence in all models of adherence, and all models of time to treatment failure. This suggests that patients who feel worse, potentially due to higher symptom side-effect profiles, or different comorbidities, seem to have worse adherence, and worse outcomes. With the findings that early treatment of HIV may result in lower transmissions [2], it would be important to study adherence in those whose health status and perceived health may be high, as one study in Uganda found that those who started ARVs at higher CD4 counts were more likely to have treatment interruptions and detectable virus at three months after initiation [21]. Female sex was associated with lower levels of non-adherence in the two bivariate models, and with self-report adherence in the multivariable model. Future studies should explore why women, in this context, might fare better than men in an ART treatment trial. The two youngest age groups had a higher hazard of treatment failure. There may be a need for additional interventions for younger individuals living with HIV/AIDS as others have documented that ART adherence can be a problem in adolescents [22]. Lastly, there may be various reasons for the findings related to region, including cultural factors related to demand characteristics (e.g. wanting to show that they are complying with study procedures, social desirability), distribution of demographic characteristics such as proportion of men and women, and clinical characteristics at baseline due to country specific treatment guidelines.

There are several limitations of the present study to note. First, although both non-adherence outcomes were associated with time to treatment failure, they are both imperfect measures. Self-report, particularly in the context of a treatment trial where those at high risk for non-adherence are not necessarily enrolled, may be influenced by demand characteristics, specifically social desirability when receiving free ART. We attempted to reduce this as much as possible using the principal components analysis to attain a non-adherence latent variable [18]. Pill count non-adherence is also imperfect in that subjects can remove pills from containers when returning to the clinic. Second, non-adherence, predictors of non-adherence, and time to failure may be different in the context of a clinical trial than in clinical care. When this study started, for example, access to ART was not widely available in all of the study settings, and hence there was high motivation to enter the study and stay in it. This may result in other demand characteristics relating to adherence. Now that ART is more widely available in global settings, particularly for those with advanced disease, it will be important to continue to study psychosocial predictors of adherence related to adherence as the epidemic and its treatment changes.

The primary finding that simplified regimens may facilitate adherence to ART is an important one for resource poor settings in that there may be a wide range of other barriers to adherence ranging from work schedule to nutritional status, and variables related to quality of life. In some locations, there are a limited number of regimens available from government sponsored programs, and hence as regimens are selected, ones with fewer requirements may be easier for patients to adhere to and therefore avoid treatment switches. The present study showed that in diverse settings, generally the simplified once a day dosing regimens had better adherence than the twice-a-day dosing over and above the effects of other important psychosocial variables, some of which also had independent effects on adherence. As newer agents are developed, the continued introduction of simplified regimens may facilitate better adherence and outcomes.
